# Mass spectrometry and multivariate analysis to classify cervical intraepithelial neoplasia from blood plasma: an untargeted lipidomic study

**DOI:** 10.1038/s41598-018-22317-6

**Published:** 2018-03-02

**Authors:** Ana C. O. Neves, Camilo L. M. Morais, Thais P. P. Mendes, Boniek G. Vaz, Kássio M. G. Lima

**Affiliations:** 1 Institute of Chemistry, Biological Chemistry and Chemometrics, Federal University of Rio Grande do Norte, Natal, 59072-970 RN Brazil; 2 School of Pharmacy and Biomedical Sciences, University of Central Lancashire, Preston, PR1 2HE United Kingdom; 3 Institute of Chemistry, Federal University of Goiás, Goiânia, 74690-900 GO Brazil

## Abstract

Cervical cancer is still an important issue of public health since it is the fourth most frequent type of cancer in women worldwide. Much effort has been dedicated to combating this cancer, in particular by the early detection of cervical pre-cancerous lesions. For this purpose, this paper reports the use of mass spectrometry coupled with multivariate analysis as an untargeted lipidomic approach to classifying 76 blood plasma samples into negative for intraepithelial lesion or malignancy (NILM, *n* = 42) and squamous intraepithelial lesion (SIL, *n* = 34). The crude lipid extract was directly analyzed with mass spectrometry for untargeted lipidomics, followed by multivariate analysis based on the principal component analysis (PCA) and genetic algorithm (GA) with support vector machines (SVM), linear (LDA) and quadratic (QDA) discriminant analysis. PCA-SVM models outperformed LDA and QDA results, achieving sensitivity and specificity values of 80.0% and 83.3%, respectively. Five types of lipids contributing to the distinction between NILM and SIL classes were identified, including prostaglandins, phospholipids, and sphingolipids for the former condition and Tetranor-PGFM and hydroperoxide lipid for the latter. These findings highlight the potentiality of using mass spectrometry associated with chemometrics to discriminate between healthy women and those suffering from cervical pre-cancerous lesions.

## Introduction

Nowadays cervical cancer is still an important issue of public health, being the fourth most prevalent type of cancer in women worldwide and accounting for an estimate of 528.000 new cases and 260.000 deaths in 2012. This situation is even worse in less developed regions, where the vast majority of new cases appear^1^. There are two main approaches to combat this particular type of cancer: i) screening programs (Pap smear, as the gold standard) and ii) more currently the human papillomavirus vaccination to protect against HPV infection. Being the most frequent sexually transmitted disease around the world^2–4^, HPV infection causes squamous intraepithelial lesions of the cervix (SIL) that may lead to cervical cancer. These pre-cancerous lesions are classified as low-grade squamous intraepithelial lesions (LSIL) and high-grade squamous intraepithelial lesions (HSIL)^5–8^ according to their potentiality to become cervical cancer over time, Once HPV infection can asymptomatically evolve to cervical cancer, it can contribute significantly to delay both diagnosis and treatment of the pre-cancerous lesions, favoring the development of the invasive disease^9,10^. In this context, new methodologies that could complement or even improve current protocols are of great interest in clinical and biomedical research. Aiming to enable early cancer diagnosis, fast, sensitive and low-cost techniques are emerging as promising alternatives for analyzing biological fluids such as blood serum/plasma and urine, which can be easily obtained from patients and reflect the pathophysiological condition of the individuals^11–14^.

Lipidomics is a sub-field of metabolomics that aims to detect and to quantify all lipids in biological samples^15^. In a more complete and biological definition, lipidomics is “the comprehensive understanding of the influence of all lipids on a biological system concerning cell signaling, membrane architecture, transcriptional and translational modulation, cell-cell and cell-protein interactions, and response to environmental changes over time”^16^. Lipids are essential biological molecules not only because they are fundamental to form the cell structure and serve as a major form of energy storage, but also because they participate in a variety of biological processes such as signaling and protein trafficking^16,17^. Recently, it has become clear that in many human diseases deregulated lipid metabolism can play an important role^17,18^. Thus lipidomics approaches can potentially help identify new mechanisms responsible for initiation of a disease process and better understand its progression to ensure that the correct treatment has been selected. Furthermore, lipidomics can promote early detection of diseases by comparing normal and diseased biological samples, considering that lipids represent key signaling molecules or biomarkers involved in physiological and disease processes^15^.

The development of soft ionization techniques has enabled mass spectrometry to become the most frequently used technique to study the metabolome/proteome of biological specimens, including those cancer-inflicted^19–23^. Via mass spectrometry, it is possible to either analyze all lipids of a sample or specific targeted lipid molecules. In untargeted lipidomics, full mass spectral profiles of lipid extracts are obtained and directly used for searching alterations in the lipid profile under biological perturbation and to discover novel or unexpected lipid metabolites^15,17^. This approach is considerably useful when comparing altered and unaltered lipid profiles. However, this strategy demands huge computational resources to convert the high complex amount of raw mass spectral data into meaningful information^17,24^. Advances in chemometrics have boosted the application of lipidomics in several research fields^25^. Multivariate analysis is a crucial tool for extracting valuable information from mass spectral data, being well-established to every step of lipidomics data analysis such as preprocessing, variable selection, metabolic identification, and modeling^25,26^. Also, chemometrics tools can help overcome some common problems of mass spectrometry related to sensitivity and reproducibility^27^.

Several recent studies have demonstrated the potentiality of mass spectrometry as a lipidomic tool for cancer research. However, most of those applications have coupled a chromatographic method with mass spectrometry to facilitate biomarkers discovery, but this methodology can be time-consuming and demands many steps of sample preparation^19,23,28^. In addition to that, chemometric methods for classification does not seem to be as widely applied in lipidomics approaches as they already are in proteomic studies^13,29,30^. Based on that, this work aims to investigate the application of an untargeted mass spectrometry lipidomics approach associated with some powerful chemometric strategies to rapidly and sensitively classify cervical pre-cancerous lesions using lipids extracted from blood plasma. The performance of some classical algorithms were compared by the ability of correctly classifying samples into NILM and SIL classes, by analyzing the obtained rates of sensitivity and specificity parameters. The chemometric modeling was based on the principal component analysis (PCA) and genetic algorithm (GA) for data reduction and variable selection, respectively; associated to linear (LDA) and quadratic (QDA) discriminant analysis, and also to support vector machines (SVM) as classification methods. Additionally, a comparison with standard classification algorithms such as k-nearest neighbors algorithm (KNN) was performed. To the best of our knowledge, this is the first study reporting the application of these algorithms in untargeted mass spectral data analysis with the proposal of classifying cervical pre-cancerous lesions in blood plasma.

## Results

### Mass spectrometry analysis and pre-processing

**Figure 1:**
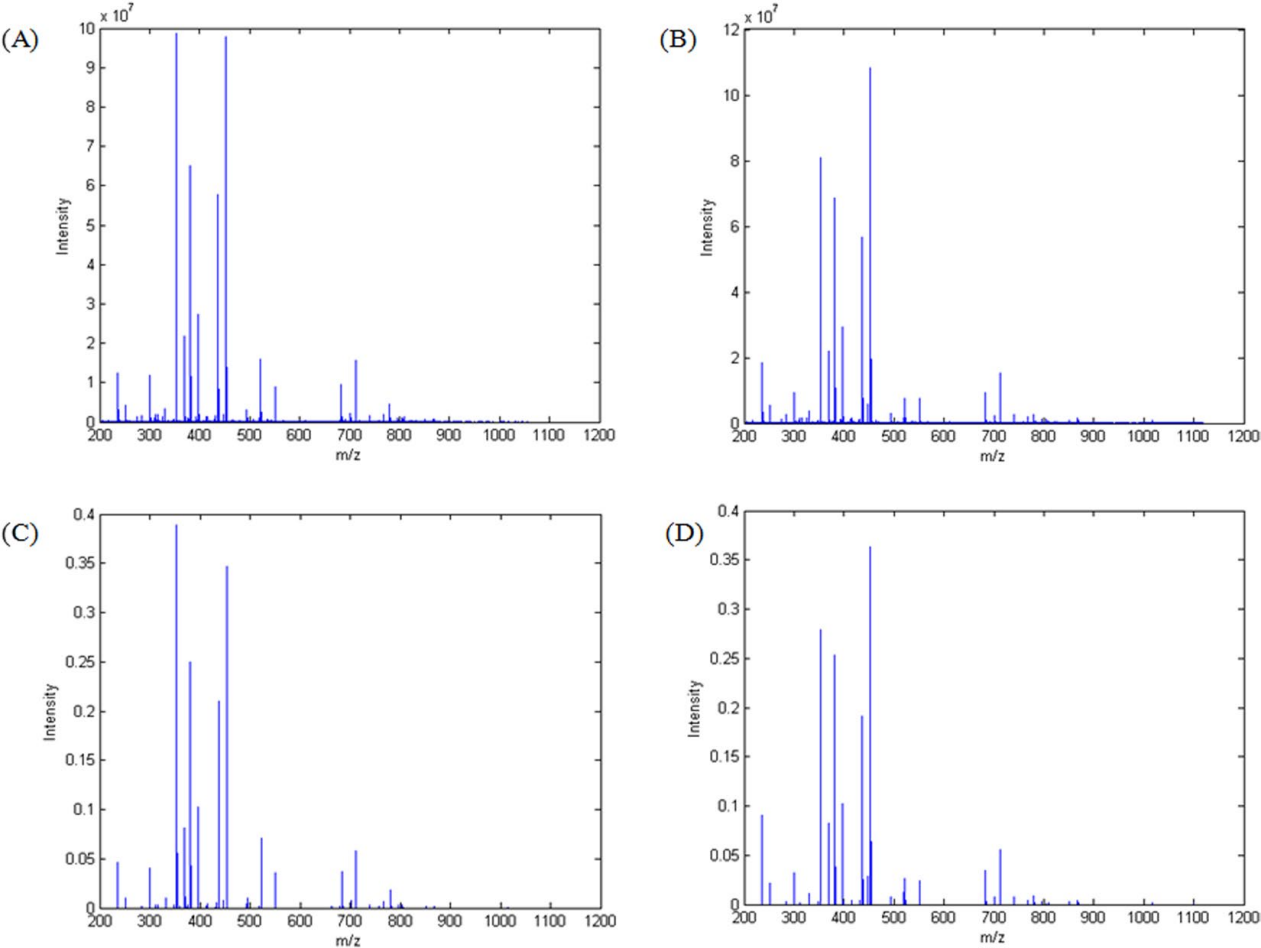
Mass spectra of metabolites extracted from blood plasma. (**A**) Mean spectrum of 42 NILM samples before pre-processing. (**B**) Mean spectrum of 34 SIL samples before preprocessing. (**C**) Mean spectrum of 42 NILM samples after pre-processing. (**D**) Mean spectrum of 34 SIL samples after preprocessing.

In this study, 76 blood plasma samples including 42 of NILM class and 34 of SIL class were subjected to lipid extraction and directly analyzed by mass spectrometry. Figure 1A and B present the mean raw spectra of NILM and SIL samples, respectively, in the *m/z* range of 200 to 1200.

To obtain meaningful information from the untargeted lipidomic data, several strategies of multivariate analysis were applied. Therefore, by using a laboratory-made routine that creates a single *m/z* vector common for all samples, all spectra were bunched into a matrix of dimension 76 × 16540 in which rows represented the 76 samples and the columns the 16540 variables (intensities of *m/z* 200–1200). Besides that, the dataset was normalized for the sum of the square of each spectrum to equals 1. Due to the high dimensionality of the matrix, a step of data compression was needed to improve computational analysis and to ensure an easier chemical interpretation. Thus, the algorithm of regions of interest (ROI) was applied to search and select only *m/z* features whose intensity were higher than a threshold of 3% of the higher intensity value, reducing the matrix dimension to 76 × 278. The mean spectra of NILM and SIL samples after normalization and peak selection are shown in Fig. 1C and D, respectively.

### Identification of lipids

**Figure 2:**
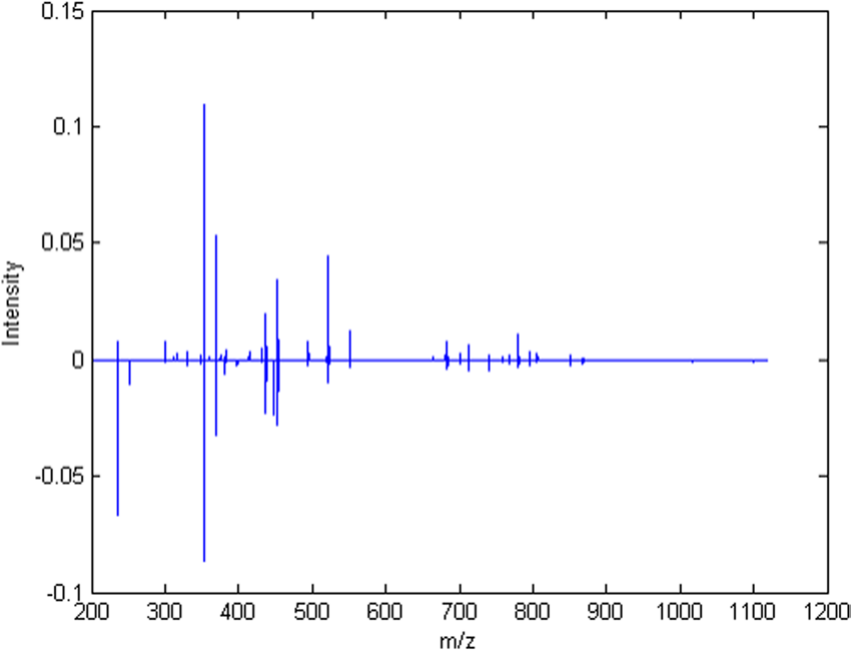
Difference between mean spectra of NILM and SIL classes.

The difference between the mean spectrum of NILM and SIL after normalization and data reduction is presented in Fig. 2. The negative signal implies that this specific *m/z* is more intense in the SIL class. Table 1 presents the main chemical information associated with the findings of Fig. 2 for mass spectrometry analysis of blood samples from both NILM and SIL patients groups. The chemical structures were proposed based on Lipid Maps Lipidomic Gateway database, and the software Xcalibur provided the errors. Five lipids were found to contribute to the distinction between NILM and SIL classes. Prostaglandins, phospholipids, and sphingolipids were associated with the NILM condition, while Tetranor-PGFM and a hydroperoxide lipid were related to the SIL class.

**Table 1:** Main chemical information associated with differentiation of NILM and SIL stages obtained from mass spectrometry analysis coupled to multivariate analysis as an untargeted lipidomic approach.

*m/z*	Error^a^	Molecular formula	Possible lipid	Class	Sample
331.177	1.540	C_16_H_27_O_7_	Tetranor-PGFM	FA^b^	SIL
369.227	0.853	C_20_H_33_O_6_	PG	FA^b^	NILM
397.258	−0.643	C_22_H_37_O_6_	HEFAD	FA^b^	SIL
680.450	0.490	C_34_H_67_O_10_NP	GPS	GPL^c^	NILM
780.526	−1.249	C_40_H_78_O_11_NS	(3′-sulfo)Galβ-Cer	SPL^d^	NILM

^a^Error in ppm; ^b^FA = Fatty acyls; ^c^GPL = Glycerophospholipids; ^d^SPL = sphingolipids.

### PCA analysis

**Figure 3:**
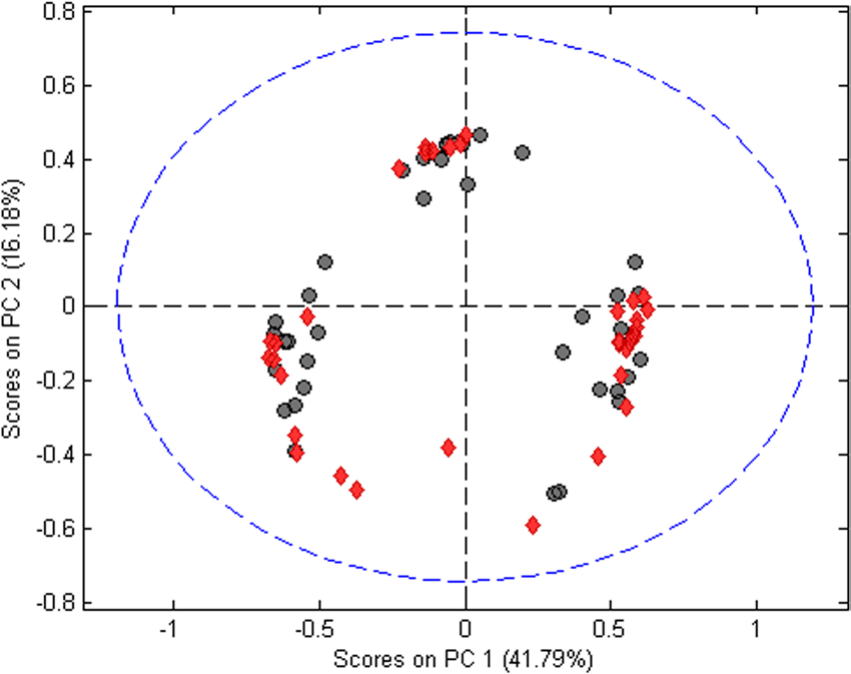
PCA scores plot for NILM (red diamonds) and SIL (gray circles) samples.

The pre-processed matrix (76 × 278) was the dataset used in all chemometric approaches. At first, an unsupervised analysis was carried out with PCA looking for trends or natural clustering behavior due to the chemical information related to the spectral signals among the samples studied. The first three principal components (PC) accounted for 64.8% of the explained variance and the scores plot for PC1 *versus* PC2 is illustrated by Fig. 3. It is possible to observe the presence of three major clusters. NILM and SIL are grouped together with no clear separation between the classes when the PC1 and PC2 area analyzed. This indicates the necessity of using more PCs for class differentiation.

### Multivariate supervised classification

Based on the results from PCA analysis, LDA, QDA, and SVM methods were applied to build classification models by using either the scores from PCA or the selected variables by GA. Table 2 presents the values of sensitivity and specificity obtained for LDA and QDA models calculated using the prediction samples. With poor correct classification rates, the ability to discriminate samples between NILM and SIL classes was not satisfactory for all LDA and QDA models. PCA-LDA/QDA (using the scores of the first ten principal components) provided the worst results where the sensitivity values varied from 0 to 60%. GA-LDA selected 22 m/z features to build a classification model that achieved better indexes of specificity and specificity than PCA-LDA. However, these values were lower than expected. On the other hand, GA-QDA selected only a small group of 15 spectral variables that did not represent properly the chemical information corresponding to the variation between the classes to correctly discriminate samples. This can be demonstrated by GA-QDA low rate of sensitivity.

**Table 2:** Results (sensitivity and specificity) of prediction samples for classifying NILM *vs*. SIL by PCA-LDA/QDA, GA-LDA/QDA and KNN.

Algorithm	Sensitivity (%)	Specificity (%)
PCA-LDA	60.0	33.3
GA-LDA	60.0	50.0
PCA-QDA	0	100
GA-QDA	40.0	83.3
KNN	60.0	66.7

Comparing these algorithms with KNN, the GA-LDA and GA-QDA had a similar performance. In terms of sensitivity, PCA-LDA, GA-LDA and KNN had the highest values (60.0%); and in terms of specificity the best was the GA-QDA (83.3%).

Table 3 presents the results achieved by the SVM models for discriminating samples into NILM and SIL classes. Sensitivity varied from 0 to 80% and specificity was better ranging from 16.6 to 100%.

**Table 3:** Sensitivity and specificity of prediction samples for classifying NILM *vs*. SIL by PCA-SVM and GA-SVM based models.

Algorithm	Sensitivity (%)	Specificity (%)
PCA-SVM-L	60.0	33.3
PCA-SVM-Q	80.0	50.0
PCA-SVM-P	80.0	83.3
PCA-SVM-RBF	80.0	83.3
PCA-SVM-MLP	20.0	16.7
GA-SVM-L	80.0	50.0
GA-SVM-Q	80.0	16.7
GA-SVM-P	40.0	66.7
GA-SVM-RBF	40.0	66.7
GA-SVM-MLP	60.0	33.3
SVM-RBF	0	100

Five different kernels were applied: linear (L), quadratic (Q), 3^rd^ order polynomial (P), radial basis function (RBF) and multilayer perceptron (MLP).

Five different Kernels were tested aiming to choose the optimal function for this study: linear (L), quadratic (Q), 3^rd^ order polynomial (P), radial basis function (RBF) and multilayer perceptron (MLP). In the same way as for the discriminant analysis, SVM classifiers were applied using the scores from PCA (of the first ten principal components) and the selected variables from GA. Sensitivity and specificity values obtained for all five GA-SVM-based models were unsatisfactory in the classification of NILM and SIL samples correctly. In all SVM models, GA selected a set of 14 spectral variables. On the other hand, PCA-SVM-based models considerably improved results, except for PCA-SVM-L and PCA-SVM-MLP that poorly separated the classes. Although PCA-SVM-Q poorly classified the NILM samples (with specificity of 50%), this model provided a satisfactory classification for samples belonging to SIL class, with sensitivity of 80%. The best performance that accurately classified samples into NILM and SIL was achieved by the models of PCA-SVM-P and PCA-SVM-RBF. These models were able to overcome the natural complexity of the data to provide sensitivity and specificity values of 80.0% and 83.3%, respectively. Comparing with SVM-RBF alone, the use of feature selection and extraction methods combined with SVM improved significantly the sensitivity.

The values of area under the curve (AUC) and F-Score for all models are shown in Table 4. For calculation, only the SVM models using RBF kernel function were considered. Among the LDA-based algorithms, the GA-LDA had the largest AUC (0.763) and F-score (0.545); and among the QDA-based algorithms, the GA-QDA was the best (AUC = 0.646 and F-score = 0.540). KNN had a performance comparable with GA-LDA and GA-QDA, having an AUC and F-score of 0.633 and 0.632, respectively. On the other hand, SVM-RBF and PCA-QDA alone had the worst performance, with an F-score of 0; therefore, no accuracy.

**Table 4:** Area under the curve (AUC) and F-Score.

Algorithm	AUC	F-score
PCA-LDA	0.536	0.428
GA-LDA	0.763	0.545
PCA-QDA	0.500	0
GA-QDA	0.646	0.540
PCA-SVM-RBF	0.817	0.816
GA-SVM-RBF	0.536	0.500
KNN	0.633	0.632
SVM-RBF	0.500	0

The algorithm with larger AUC and F-Score was the PCA-SVM-RBF, with values of 0.817 and 0.816, respectively. This confirms that PCA-SVM-RBF is the best model, having the highest probability of correctly classify a randomly positive sample and also the most accurate one.

**Figure 4:**
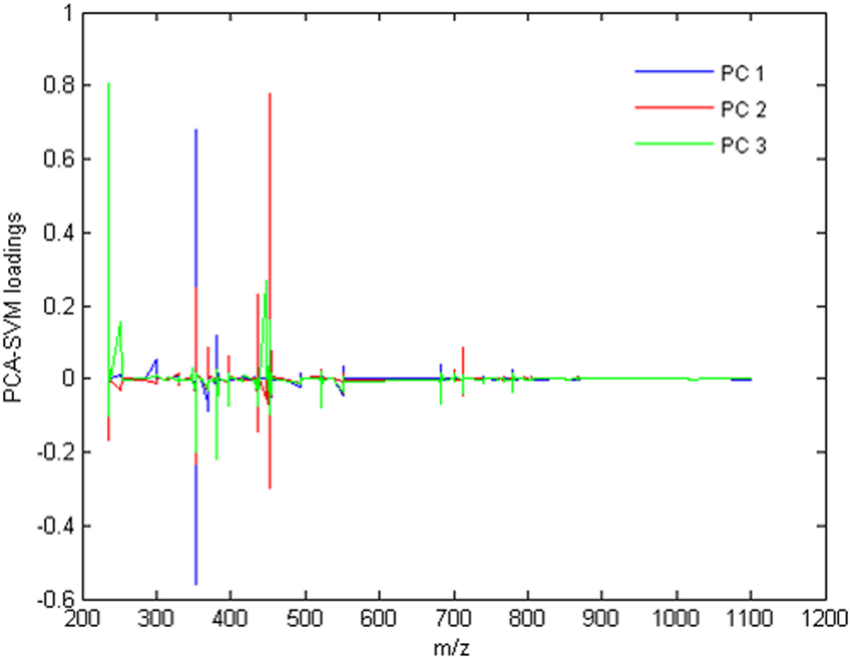
PCA-SVM-RBF loadings on PC1 (blue), PC2 (red) and PC3 (green).

Figure 4 shows the loadings of PC1, PC2 and PC3 for the PCA-SVM-RBF model.

Taking into account that the coefficients can be understood as the influence of the variables (*m/z* features) for the PCA-SVM-RBF model performance, for the three principal components the most important variables for class differentiation are present in the region around *m/z* 200 to 450 as also, but less intensely, in the range of *m/z* 700 to 800. These loadings are consistent with the regions that have the highest concentration of m/z signals for all samples, which consequently matches the region where the main spectral differences between NILM and SIL samples are found.

## Discussion

Considering that lipids play an important role in metabolism, either in physiological or disease condition, an untargeted metabolomic approach was applied to classify samples according to spectral changes that could be attributed to the pathophysiological condition of the individuals. From Figs 1 and 2, it is possible to note that the majority of the signals are at low *m/z* range for both classes, giving them high spectral similarity, what makes it difficult to identify the classes only visually. There are only smooth differences when comparing the two classes, once again reinforcing the use of multivariate analysis to discriminate NILM and SIL samples. Since mass spectrometry experiments were performed without the assistance of a chromatographic separation technique and due the inherent ionization suppression events observed on ESI, it was not possible to characterize a high number of chemical species in both NILM and SIL classes within acceptable errors.

The peak at *m/z* 331.177 was attributed to the prostaglandin tetranor-PGFM, a metabolite of the PGF_2α_^31^ associated with acute inflammation conditions^32,33^. The signal at *m/z* 369.227 can be related to several prostaglandin metabolites (PGM) such as PGG2, 6-keto PGE1, 20-hydroxy-PGE2, among others. Furthermore, this *m/z* feature is also correlated to Tromboxane B2 and B3 as well as other eicosanoic acid derivatives. There are hundreds of distinct arachidonic acid (as well as other fatty acids) derivatives described and properly characterized as eicosanoids that play relevant roles as bioactive signaling lipids, including regulation of several homeostatic and inflammatory processes^34^. The peak at *m/z* 397.258 is supposedly associated with a hydroperoxide epidioxide fatty acid derivative (HEFAD). Natural oxidation of lipids has a relevant role in many biological processes, including those associated with the development of diseases such as cancer^35^. In fact, the literature reports lipid hydroperoxides as major primary products and able to undergo several secondary reactions, including their role as substrates in enzymatic reactions^36^. The peak at *m/z* 680.455 may be correlated to a serine-based glycerophospholipid (GPS). In fact, it is well established that phospholipids play a crucial role in cell structure as main constituents of the membranes among other primordial functions and applications^37^. Lastly, the signal at *m/z* 780.526 is related to (3′-sulfo)Galβ-Cer, an sphingolipid of the sulfatides subclass. Sulfatides are found in several parts of the human body and comprehend multifunctional chemical species that play an important role in many biological functions, including those associated, for example, with nervous and immune systems, including health and disorder conditions^38^.

The findings presented above indicate that prostaglandin tetranor-PGFM as well as HEFAD are related directly to SIL conditions in the patients of this study. The literature supports the role of inflammatory pathways leading to abnormal production of prostaglandins that can be directly associated with cervical cancer, which is coherent for the former lipid^39^. On the other hand, enhanced lipid peroxidation levels are associated with oxidative injuries and cervical cancer cases when compared to healthy patients^40^.

Lipids are metabolites that can extremely vary between individuals and in non-disease/disease conditions. Furthermore, the plasma samples used in this study have come from women with considerably different lifestyle, ages, habits, weights and diseases/medical treatments that are all factors that strongly influence both the lipid amount and composition present in the bloodstream. All that information which is not directly related to cervical pre-cancerous lesions can be contemplated by the untargeted lipid extraction, adding extra complexity to classify NILM and SIL samples. In addition to that, there is an intrinsic source of variation related to SIL class due to the existence of two sub-classes LSIL and HSIL among the samples, what can also contribute to enhancing variation within SIL class to the detriment of the variation between the classes NILM and SIL, impairing the classification performance. Given the complexity observed for the classification based on discriminant analysis models (LDA and QDA), a strategy of using a non-linear supervised method was adopted, and for that purpose, the SVM algorithm was applied. SVM is a powerful tool for dealing with biological data since biological processes commonly follow a non-linear response^25^.

SVM-RBF loadings support the chemical meaning of the multivariate statistical model, suggesting that five lipids are involved in the spectral changes that allow for differentiation between blood plasma of healthy women (NILM) and those suffering from cervical pre-cancerous lesions (SIL). It is worth mentioning that the ability to classify NILM and SIL samples with sensitivity and specificity values around 80.0% and 83.3% may represent significant clinical interest, since the classification rates obtained by this proposed methodology can be compared to those provided by traditional Pap smear test, with the advantage of avoiding the inner subjectivity of the cytological method.

In conclusion, the results obtained in this study present the potentiality of mass spectrometry associated with multivariate analysis as a promising alternative to classifying blood plasma of NILM and SIL women based on an untargeted lipidomic approach. The experimental methodology was simple, fast, and with minimal sample treatment, being directly injected in the mass spectrometer. Despite the natural complexity of lipids exhibit in biological organisms, multivariate analysis was able to extract meaningful information from mass spectrometry data allowing to classify the analyzed samples correctly. The best model, PCA-SVM with RBF kernel, achieved very satisfactory values of sensitivity (80%) and specificity (83.3%), and a high AUC (0.817) and F-score (0.816) indicating its good predictive capacity and accuracy. The utilization of PCA and GA algorithms for data reduction contributed to simplify the chemical interpretation of results and to speed up computational analysis, since a set of 278 m/z features could be reduced to ten principal components or less than twenty selected variables by GA. In addition, the predictive performance of SVM models combined with PCA and GA was higher than using SVM alone. Some lipid spectral changes were suggested to be contributing to differentiation of samples related to NILM and SIL conditions. While PG, GPS and (3′-sulfo)Galβ-Cer are related to NILM class, Tetranor-PGFM and HEFAD are directly associated with SIL condition. While these results are encouraging, much larger databases of mass spectra of a wider range of medical data, as well as a larger number of blood plasma samples, must be established in order to both look for possible biomarkers of cervical pre-cancerous disease and also to study the ability of the proposed analysis, based on direct mass spectrometry combined to chemometrics tools, with a view to make it possible to properly include this proposed methodology as an efficient alternative in clinical routine situations.

## Methods

### Collection and preparation of specimens

Women living in the state of Rio Grande do Norte/Brazil attending the Maternidade Escola Januário Cicco (MEJC) of the Public Health System for cervical pathology screening consultations and reference services for colposcopy were volunteers in this study during July 2014 to March 2016. The Institutional Ethics Committee for Human Research of the Hospital Universitário Onofre Lopes (HUOL), of the Federal University of Rio Grande do Norte (UFRN), Brazil, approved this study (protocol #526/11) and informed consent was obtained from all subjects. Also, all the methods carried out in this study were by the approved guidelines. A total of 76 blood samples of different women were collected by venipuncture in tubes containing the anticoagulant EDTA and within two hours after blood collection plasma was separated by density gradient, and aliquots were transferred into cryogenic tubes and stored at −80 °C until analysis. Right after blood collection, women were submitted to cytology smears or large loop excision surgery of the transformation zone (LLETZ). For specimens obtained from LLETZ, histopathological analysis was performed on sections from paraffin blocks in 4 μm thickness and stained with hematoxylin/eosin. Cytology and histopathology were reported according to the Bethesda System^41^: 42 patients (NILM) and 34 patients (SIL), where 13 are LSIL and 31 are HSIL.

### Lipid extraction

The Folch method was used for lipid extraction, based upon Patterson *et al*.^42^. The plasma samples were prepared with one aliquot of 40 µL of blood plasma. Next, 160 µL of methanol was added to samples followed by 320 µL of chloroform. Samples were vortexed and incubated on ice for 20 min. Samples were vortexed again before the addition of 150 µL of water to induce phase separation and incubation on ice for another 10 min. Folch samples were then centrifuged for 5 min at 10,000 rpm. The bottom layer (organic) was removed to a new, clean microcentrifuge tube before the top layer was reextracted with 250 µL of chloroform: methanol (2:1, v/v). The extraction was vortexed and centrifuged during additional 5 min. Next, the samples were dried. Finally, they were reconstituted in 200 µL isopropanol and transferred to vials before MS analysis.

### Mass spectrometry conditions

Samples were analyzed in an Electrospray ionization (ESI) coupled to Q Exactive Orbitrap mass spectrometer (Thermo Scientific, Bremen, Germany). The mixture was directly infused into the ESI source at a flow rate of 5 μL min^−1^. Mass spectra were recorded in full MS mode ranging from *m*/*z* 200 to 1200 in positive mode. Other experimental parameters used were: spray voltage of 3.0 kV; capillary temperature of 250 °C; S-Lens RF level of 50%; average of 3 micro-scans for each spectrum; and resolution of 140,000. The spectra were processed by the Xcalibur Analysis software package (version 2.0, Service Release 2, Thermo Electron Corporation).

### Identification of lipids

The chemical structures were proposed based on LIPID MAPS® Lipidomic Gateway database^43^ following the way: → ‘Tools’ → ‘Search the LMSD for lipids with a given mass (m/z) value. Display structure and isotopic distribution profile’. Errors were provided by Thermo Scientific^™^ Xcalibur software and they were calculated by the difference between the theoretical mass and the experimental mass.

### Data analysis and chemometric methods

Computational analysis including import and pre-processing of data as well as construction of multivariate classification models (PCA-LDA, PCA-QDA, PCA-SVM, GA-LDA, GA-QDA, GA-SVM, KNN and SVM) were performed within MATLAB® R2010a environment (MathWorks Inc, Natick, MA, USA) by using PLS Toolbox version 7.9.3 (Eigenvector Research, Inc., USA) and laboratory-made routines. Initially, to bunch all spectra into a regular data matrix, i.e. samples in the rows and m/z intensities in the columns, it was necessary to create a common vector of *m/z* values, since each sample had its own mass spectrum (with different values of *m/z*). Thus, a laboratory-made routine was employed to guarantee that all mass spectra had the same size, then the data matrix of dimensions 76 × 16540 was built. After that, the matrix was normalized so that the sum of squares of each row (sample) was equals to 1. The final pre-processing step was the compression (dimensionality reduction) of the data matrix, and for that, it was applied the strategy of selection of mass spectrometry regions of interest (ROIs), adapted to analyze only mass spectra (chromatographic free) as input data. ROIs allows the selection of *m/z* values whose intensity signals are higher than a determined threshold value (in this case, 3% of the maximum intensity value). A more detailed description of ROIs can be appreciated elsewhere^44^. The compressed matrix, after ROIs selection, was considerably reduced to the dimension of 76 × 278, and this matrix was subsequently used as inputs for the classification models.

Before the modeling, samples were divided into training (70%, 54 samples), validation (15%, 11 samples) and prediction (15%, 11 samples) sets by the classical Kennard-Stone (KS) algorithm^45^. Model construction and optimization (variable selection by GA) was carried out using the training samples; and the validation set was applied to test its internal performance. The left out samples, the prediction set, were applied to evaluate the classification accuracy by LDA, QDA and SVM discrimination approaches. The pre-processed data was applied to the classification algorithms in two steps: first, data reduction was carried out by PCA or GA; then the scores obtained by PCA and the spectral variables selected by GA were utilized as input data for LDA, QDA and SVM classification methods. KNN was performed with the whole mass spectra using the validation set to determine the k-value. The best result was found with k = 3.

For variable selection using GA, the optimal number of variables was achieved by minimizing the average risk of misclassification G, calculated in the validation set as:

1 $$G=\frac{1}{{N}_{V}}\sum _{n=1}^{{N}_{v}}{g}_{n}$$

where *N*_*V*_ is the number of validation samples; and *g*_*n*_ is defined as,

2 $$G=\frac{{r}^{2}({x}_{n},{m}_{I(n)})}{{{\rm{\min }}}_{I(m)\ne I(n)}{r}^{2}({x}_{n},{m}_{I(m)})}$$

where *I*(*n*) is the index of the true class for the *n*th validation object x_n_; ${r}^{2}({x}_{n},{m}_{I(n)})$ is the squared Mahalanobis distance between object x_n_ (of class index *I*(*n*)) and the sample mean ${{\rm{m}}}_{{\rm{I}}({\rm{n}})}$ of its true class; and ${{\rm{r}}}^{2}({{\rm{x}}}_{{\rm{n}}},{{\rm{m}}}_{{\rm{I}}({\rm{m}})})$ is the squared Mahalanobis distance between object *x*_*n*_ and the center of the closest wrong class^46^. The minimum value of the cost function (maximum fitness) will be achieved when the selected variables from the original data are closer as possible to its true class and more distance as possible from its wrong class according to the validation samples. GA calculations were performed through 40 generations having 80 chromosomes each. The risk of overfitting with GA was reduced by setting the crossover probability to a relatively large number (60%) in order to increase the size of the offspring due to the small number of samples; and the mutation probability was set to a relatively large value (10%) so the model could adjust to a better fitting throughout mutation. Further, the algorithm was repeated three times, starting from different random initial populations. The best solution (in terms of the fitness value) resulting was employed.

LDA classification score (*L*_*ik*_) is calculated for a given class *k* by the following equation in order to obtain a discriminant profile:

3 $${L}_{ik}={({{\bf{x}}}_{i}-{\bar{{\bf{x}}}}_{k})}^{{\rm{T}}}{{\boldsymbol{\Sigma }}}_{{\rm{pooled}}}^{-1}({{\bf{x}}}_{i}-{\bar{{\bf{x}}}}_{k})-2{\mathrm{log}}_{e}{\pi }_{k}$$

where x_1_ is an unknown measurement vector for sample *i*; ${\bar{{\rm{x}}}}_{{\rm{k}}}$ is the mean measurement vector of class *k*; ${{\boldsymbol{\Sigma }}}_{{\rm{pooled}}}$ is the pooled covariance matrix; and π_k_ is the prior probability of class *k*^47^.

QDA classification score (Q_ik_) is estimated using the variance-covariance for each class *k* and an additional natural logarithm term, as follows:

4 $${Q}_{ik}={({{\bf{x}}}_{i}-{\bar{{\bf{x}}}}_{k})}^{{\rm{T}}}{{\boldsymbol{\Sigma }}}_{k}^{-1}({{\bf{x}}}_{i}-{\bar{{\bf{x}}}}_{k})+{\mathrm{log}}_{e}|{{\boldsymbol{\Sigma }}}_{k}|-2{\mathrm{log}}_{e}{\pi }_{k}$$

where ${{\boldsymbol{\Sigma }}}_{k}$ is the variance-covariance matrix of class *k*; and ${\mathrm{log}}_{{\rm{e}}}|{{\rm{\Sigma }}}_{{\rm{k}}}|$ is the natural logarithm of the determinant of variance-covariance matrix of class *k*. QDA forms a separated variance model for each class and does not assume classes having similar variance-covariance matrices, differently of what is assumed by LDA^48^.

SVM classification essentially consists in nonlinearly mapping the original data into a much higher dimensional feature space using a Kernel function, and then constructing an optimal hyperplane that separates objects of two classes maximizing the margins of separation^49,50^. Kernel functions appear in various types (linear, quadratic, polynomial, radial basis function, among others) and their applications change the classification ability of SVM^25^. In this study, different kernel functions were utilized, and they are calculated as follows:

Linear,

5 $$k({{\bf{x}}}_{i},{{\bf{z}}}_{j})={{\bf{x}}}_{i}^{{\rm{T}}}{{\bf{z}}}_{j}$$

Quadratic,

6 $$k({{\bf{x}}}_{i},{{\bf{z}}}_{j})={(\tau +{{\bf{x}}}_{i}^{{\rm{T}}}{{\bf{z}}}_{j})}^{2},\,\tau \ge 0$$

3^rd^ order polynomial,

7 $$k({{\bf{x}}}_{i},{{\bf{z}}}_{j})={(\tau +{{\bf{x}}}_{i}^{{\rm{T}}}{{\bf{z}}}_{j})}^{3},\,\tau \ge 0$$

Radial basis function (RBF),

8 $$k({{\bf{x}}}_{i},{{\bf{z}}}_{j})=\exp (-\gamma \Vert {{\bf{x}}}_{i}-{{{\bf{z}}}_{j}}^{2}\Vert )$$

Multilayer perceptron (MLP),

9 $$k({{\bf{x}}}_{i},{{\bf{z}}}_{j})=\,\tanh ({k}_{1}{{\bf{x}}}_{i}^{{\rm{T}}}{{\bf{z}}}_{j}+{k}_{2})$$

where **x**_*i*_ and **z**_*j*_ are sample measurements vectors; *τ* is a constant; *γ* is a tuning parameter that controls the RBF width; and *k*_1_ and *k*_2_ are constants. Finally, the SVM classifier is obtained by the following decision function^49,50^:

10 $$f(x)={\rm{sign}}(\sum _{i=1}^{{N}_{SV}}{\alpha }_{i}{y}_{i}k({{\bf{x}}}_{i},{{\bf{z}}}_{j})+b)$$

where *N*_*SV*_ is the number of support vectors; α_i_ is the Lagrange multiplier; y_i_ is the class membership (±1); ${\rm{k}}({{\rm{x}}}_{{\rm{i}}},{{\rm{z}}}_{{\rm{j}}})$ is the kernel function; and *b* is the bias parameter.

In the case of clinically classifying non-disease (NILM) and disease (SIL) samples, sensitivity can be understood as the probability that a test result will be positive when the disease is present, while specificity is the probability that a test result will be negative when the disease is not present. To statistically evaluate the classification models, calculations of sensitivity and specificity were performed using the test samples as important quality measures of model accuracy. Both parameters have a maximum value of 100 and a minimum of 0, and are obtained as follows:

11 $${\rm{Sensitivity}}( \% )=\frac{{\rm{TP}}}{{\rm{TP}}+{\rm{FN}}}\times 100$$

12 $${\rm{Specificity}}( \% )=\frac{{\rm{TN}}}{{\rm{TN}}+{\rm{FP}}}\times 100$$

where FN is defined as a false negative and FP as a false positive; and TP and TN are defined as true positive and true negative, respectively.

Also, the models were evaluated using the area under the curve (AUC) and F-score. The AUC is the area under the receiver operating characteristics conditions (ROC) curve, and the F-score is a measurement of the model accuracy defined by:

13 $$F-score=\frac{2\times SENS\times SPEC}{SENS+SPEC}$$

where SENS stands for sensitivity; and SPEC stands for specificity.
